# Supplementary material for: Bufalin Suppresses Head and Neck Cancer Development by Modulating Immune Responses and Targeting the β-Catenin Signaling Pathway
Source: Cancers (Basel). 2024 Aug 1;16(15):2739. doi: 10.3390/cancers16152739 (PMC11311268; doi:10.3390/cancers16152739)

Figure 2B : Bufalin's effects on cancer cells apoptosis. Western Blot analysis of apoptosis markers following bufalin incubation for various times, ranging from 1 hour to 48 hours, in both cell lines.

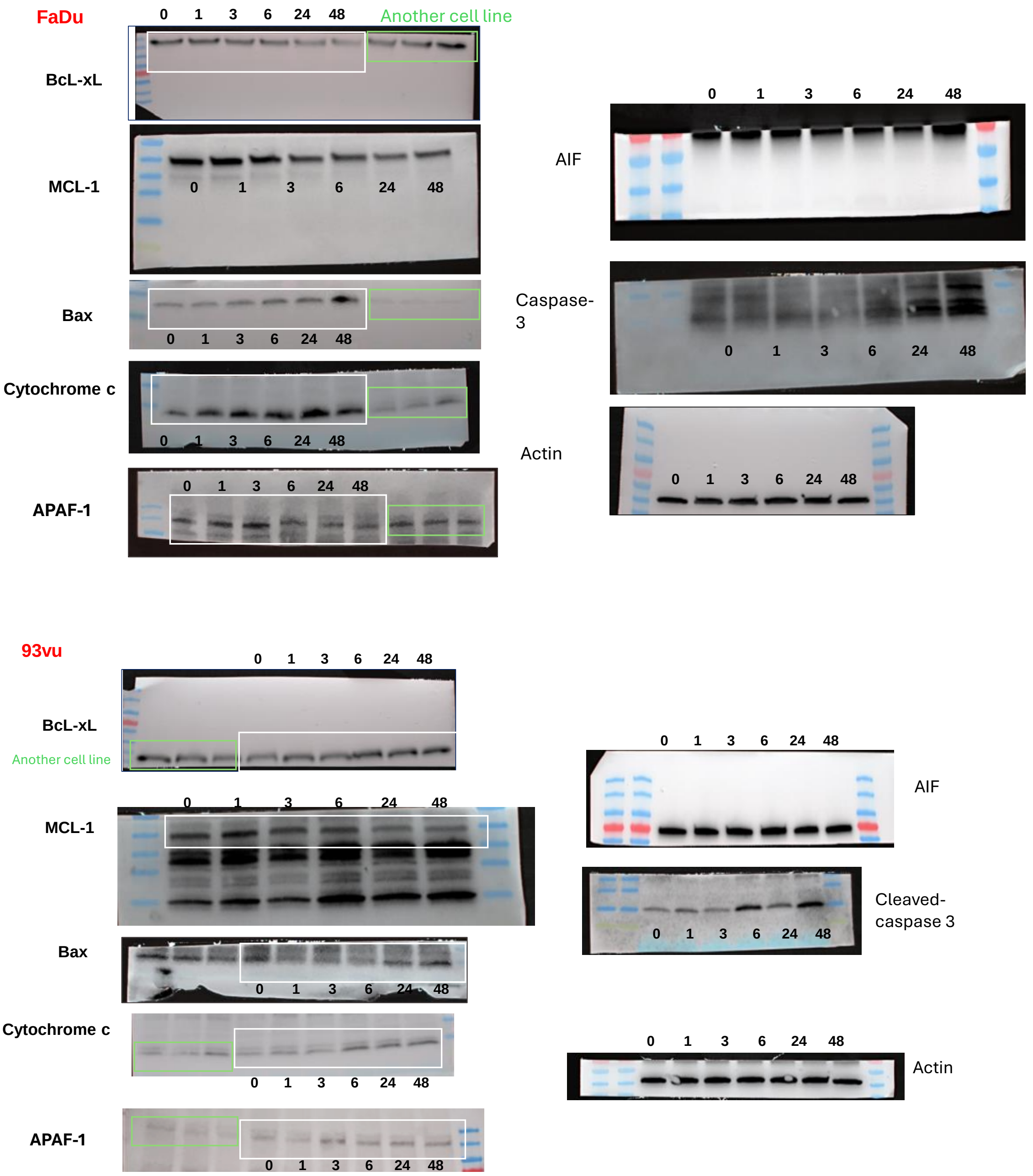

Figure 3B : Bufalin's effects on oxidative stress. Western Blot analysis of NRF2 marker following 24 hours of bufalin IC_{80} incubation in both cell lines.

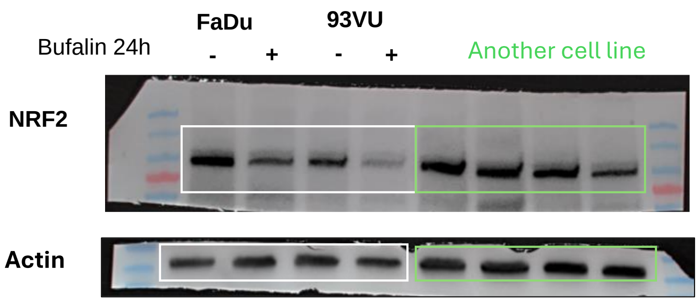

Figure 4A. Bufalin effects on cell cycle regulation. Western Blot analysis of cell cycle-related markers following cell incubation with bufalin IC_{80} for various times, ranging from 1 hour to 48 hours.

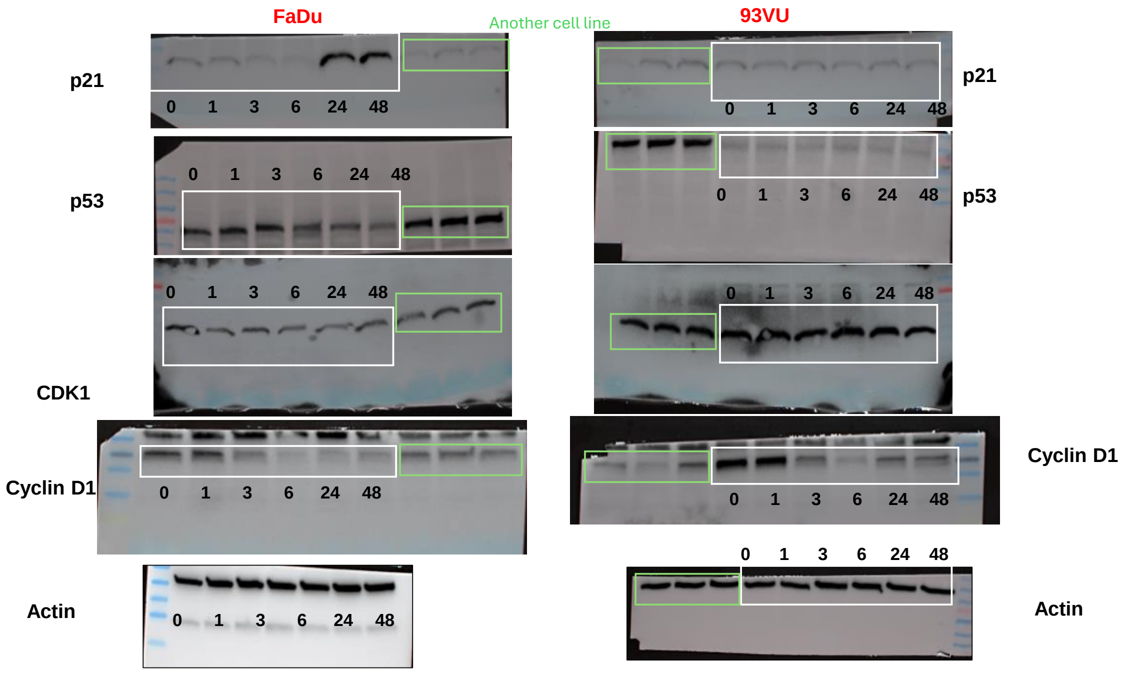

Figure 6A. Bufalin downstream different pathways. Western Blot analysis showing the expression levels of p-β-catenin (Ser675), total β-catenin, EGFR and p-STAT3 (Tyr605) bufalin treatment for various durations, ranging from 1 to 48 hours.

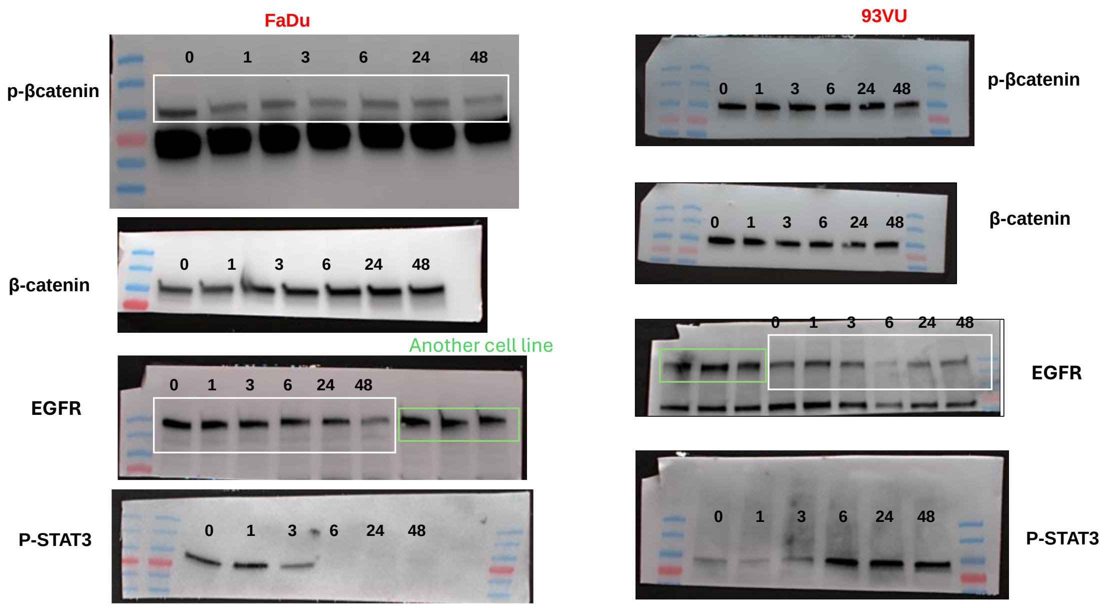

Figure 6B. Western blot analysis of the proto-oncogene c-Myc marker following 24 hours of bufalin incubation in both cell lines.

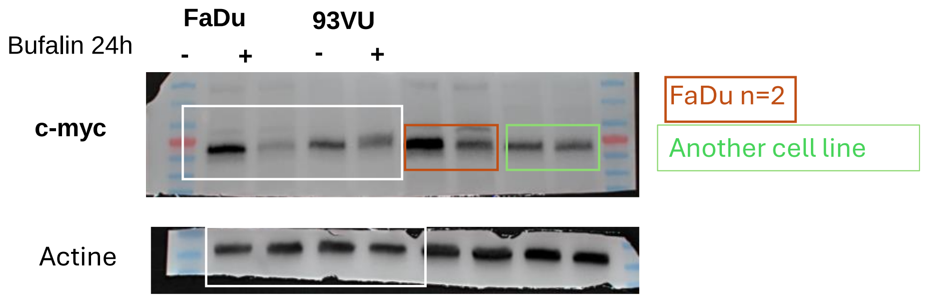

Supplement: Supplementary file 1 [file cancers-16-02739-s001.zip › cancers-3101247-supplementary.pdf]
